# Clinical efficacy of neoadjuvant chemotherapy regimens FLEEOX vs. XELOX in patients with initially unresectable advanced gastric cancer: a propensity score analysis

**DOI:** 10.18632/oncotarget.19004

**Published:** 2017-06-28

**Authors:** Yang Li, Jun Chen, Qi He, Xiang Ji, Xulin Wang, Chaogang Fan, Guoli Li

**Affiliations:** ^1^ Division of Digestive Surgery, Xijing Hospital, Fourth Military Medical University, 710032, Xi’an, Shaanxi, China; ^2^ Research Institute of General Surgery, Jinling Hospital, School of Medicine, Nanjing University, Nanjing 210002, China

**Keywords:** initially unresectable advanced gastric cancer, neoadjuvant chemotherapy, tumor response rate, overall survival, chemotherapy-related toxicity

## Abstract

**Purpose:**

This study was designed to assess the effectiveness of FLEEOX (5-Fu, leucovorin, etoposide, oxaliplatin, and epirubicin) compared with XELOX (capecitabine and oxaliplatin) as neoadjuvant chemotherapy (NAC) for initially unresectable advanced gastric cancer (AGC).

**Methods:**

This study reviewed patients who underwent FLEEOX or XELOX for initially unresectable AGC. To reduce the bias in patient selection, we conducted propensity score match (PSM) with 1:1 ratio. Tumor and pathological response, surgical characteristics, chemotherapy-related toxicity and overall survival (OS) were analyzed.

**Results:**

From January 2004 to December 2012, 436 patients were enrolled; 99 pairs of patients were generated after PSM. The tumor response rates were 80.8% and 68.7% in FLEEOX and XELOX (*P*=0.018). 80 patients (80.8%) in FLEEOX and 63 (63.6%) in XELOX received radical resection (*P*<0.001). The pathological complete response rate and R0 rate were 11.1% and 69.7% in FLEEOX, respectively, while 4.8% and 38.4% in XELOX (*P*<0.001). Median OS time was longer in FLEEOX (30.0 vs. 25.1 months, *P*<0.001). In addition, more toxicities occurred in FLEEOX, including leukocytopenia (17.2% vs. 7.1%, *P*=0.024), nausea (17.2% vs. 6.1%, *P*=0.012) and vomiting (22.2% vs. 10.1%, *P*=0.016). The overall toxicity rate was higher in FLEEOX (71.7% vs. 35.4%, *P*<0.001).

**Conclusion:**

The FLEEOX regimen as NAC for patients with initially unresectable AGC can improve tumor response rate, radical resection rate, R0 rate, and OS as compared to XELOX regimen. More chemotherapy-related toxicity was observed in FLEEOX group, although no chemotherapy-related deaths and aborting were observed. Further randomized clinical trials on the FLEEOX regimen are necessary.

## INTRODUCTION

Gastric cancer is one of the leading cause of cancer-related deaths worldwide [1] and causes 697,000 new diagnosed cases and 499,000 related deaths in China each year [2]. Moreover, 80%-90% gastric cancer patients in China are diagnosed at an advanced stage with extensive regional lymph node involvement or invasion of adjacent structures in first medical consultation [3, 4]. Additionally, only 50-60% of gastric cancer patients are suitable candidates for curative surgery [5]. When radical resection cannot be done at first diagnosis, the prognosis of patients with advanced gastric cancer (AGC) is rather poor [6].

Neoadjuvant chemotherapy (NAC) is a promising strategy of multimodality therapies. It is currently accepted as an effective treatment for ovarian, head and neck cancer and extremity tumors, considered to have many clinical advantages [7]. For potentially resectable patients, exposure to chemotherapy at the earliest time may prevent rapid growth of metastases after therapy of the primary sites and may also prevent the emergence of chemoresistant clones. Moreover, several studies have indicated that initially unresectable AGC can be successfully converted to resectable AGC by NAC and then treated with curative surgery [8–11]. However, the NAC regimens were various in these trials. S-1 plus cisplatin has been reported with 63% response rate (RR) and 31.0% 3-year overall survival (OS) rate in initially unresectable AGC in a phase II clinical trial [12]. Further studies indicated that the addition of docetaxel to cisplatin and S-1 could improve the outcome of patients with unresectable gastric cancer [13, 14]. In contrast [15], the addition of epirubicin to XELOX (capecitabine and oxaliplatin) didn’t improve effects in RR, radical resection rate, and OS benefits. And the chemotherapy-related toxicity caused by this triple-drug regimen was higher. So, the clinical efficacy of these chemotherapy regimens and whether multi-drug regimens bring more benefits than double-drug therapies do for these patients remain unknown.

So, we intended to find an easy and effective approach which can permit the use of a multi-drug regimen offering more effectiveness and decrease the chemotherapy-related toxicity simultaneously. The research conducted by Nakajima et al. gave a clue, in which the intra-arterial approach was used to inject etoposide and cisplatin for the treatment of AGC patients with excellent efficacy and reduced toxicity [16]. Thus, based on the same theory, we developed an NAC regimen (FLEEOX: 5-FU, leucovorin, etoposide, oxaliplatin, and epirubicin) via intra-arterial and intravenous administration for patients with initially unresectable AGC. In our previous research, it has been demonstrated that FLEEOX could produce a favorable tumor RR with relatively mild toxicity profile for AGC patients with para-aortic lymph nodal metastasis [17]. In addition, fluorouracil plus oxaliplatin is a recommended two-drug regimen for AGC patients according to the National Comprehensive Cancer Network (NCCN) Guideline Edition 2007, and the fluorouracil can be replaced with capecitabine. Therefore, the aim of this study was to evaluate the clinical efficacy of FLEEOX for patients with initially unresectable AGC compared with XELOX (capecitabine and oxaliplatin) by means of a case-matched study with propensity score matching (PSM). Besides, the chemotherapy-related toxicity was also analyzed.

## RESULTS

### Patients

**Figure 1:**
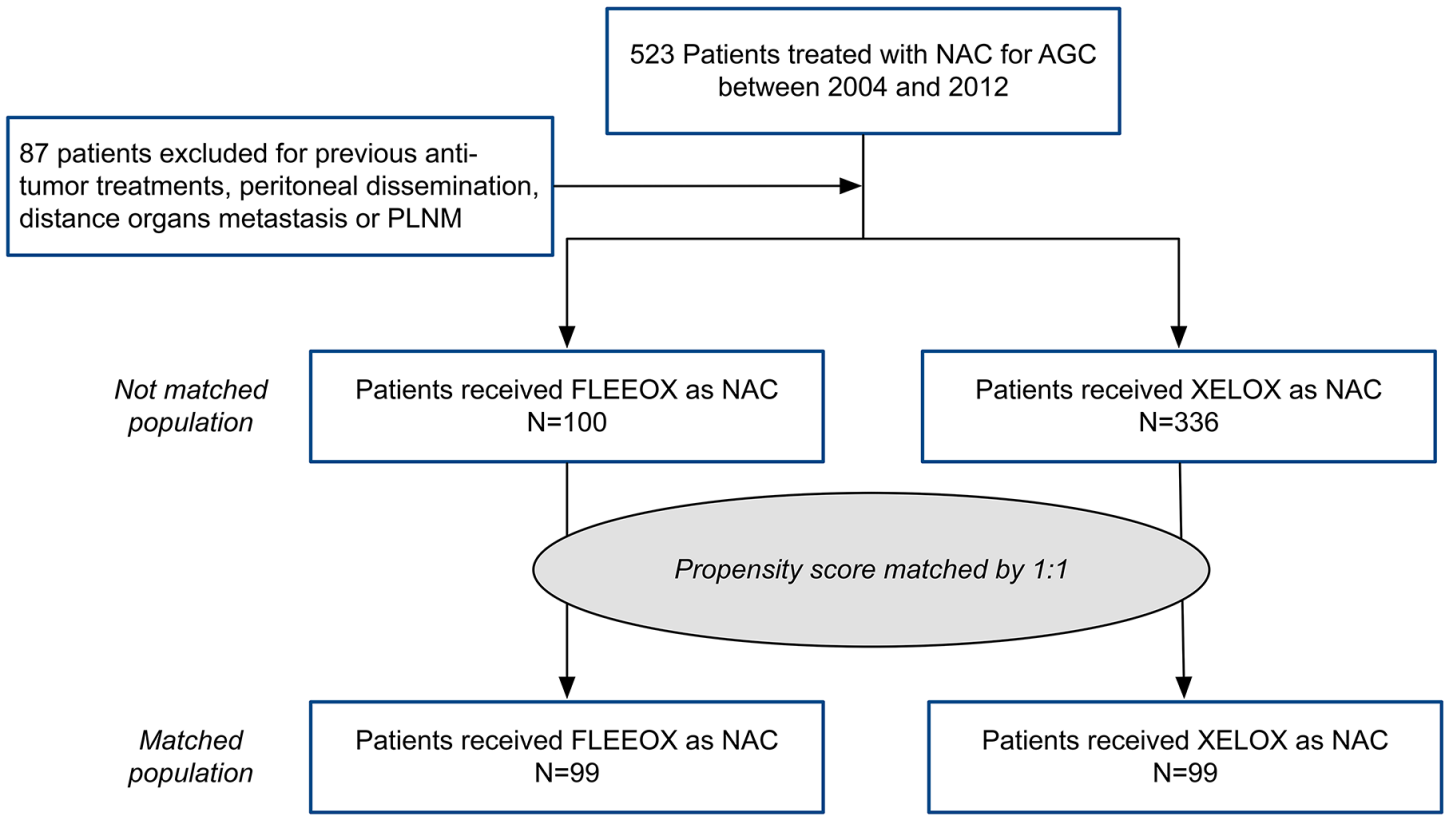
Patient study group CONSORT diagram

From January 2004 to December 2012, a total of 523 patients receiving NAC for AGC in our institution were reviewed (Figure 1). Of them, 87 patients were excluded because of receiving other anti-tumor treatments, peritoneal dissemination, distance organs metastasis or para-aortic lymph nodal metastases (PLNM). The remaining 436 patients who underwent either FLEEOX (n=100) or XELOX (n=336) were eligible for inclusion in this research.

### Baseline characteristics

The baseline characteristics before and after PSM were listed in Table 1. Before the matching, there were significant differences in gender, tumor location, differentiation, and cause of unresection between FLEEOX and XELOX groups (*P*<0.001), whereas the clinical T and N classification were similar. The disparities were resolved, however, after the PSM performed, and no differences (*P*>0.05) in the baseline characteristics were observed between the two matched groups.

**Table 1:** The characteristics of the patients before and after PSM

Variable	Before PSM			After PSM		
	FLEEOX (n=100) (%)	XELOX (n=336) (%)	*P*	FLEEOX (n=99) (%)	XELOX (n=99) (%)	*P*
Age (mean years±SD)	60.89±10.37	59.99±9.19	0.357	60.98±10.43	59.74±9.54	0.383
Gender			**<0.001**			0.126
Male	70 (70.0)	294 (87.5)		70 (70.7)	78 (78.8)	
Female	30 (30.0)	42 (12.5)		29 (29.3)	21 (21.2)	
Tumor location			**<0.001**			0.583
Upper third	20 (20.0)	114 (32.1)		26 (26.3)	23 (23.2)	
Middle third	54 (54.0)	181 (53.9)		54 (54.5)	61 (61.6)	
Lower third	26 (26.0)	61 (14.0)		19 (19.2)	15 (15.2)	
Differentiation			**<0.001**			0.151
High-medium	26 (26.0)	111 (33.0)		26 (26.3)	23 (23.2)	
Low	73 (73.0)	160 (47.6)		72 (72.7)	70 (70.7)	
Undifferentiating	1 (1.0)	65 (19.3)		1 (1.0)	6 (6.1)	
Clinical T classification			0.450			0.387
cT3	40 (40.0)	139 (41.4)		40 (40.4)	43 (43.4)	
cT4	60 (60.0)	197 (58.6)		59 (59.6)	56 (56.6)	
Clinical N classification			0.369			0.257
cN1	24 (24.0)	72 (21.4)		24 (24.2)	22 (22.2)	
cN2	39 (39.0)	113 (33.6)		38 (38.4)	29 (29.3)	
cN3	37 (37.0)	151 (44.9)		37 (37.4)	48 (48.5)	
Cause of unresection			**<0.001**			0.173
Local advance	15 (15.0)	132 (39.3)		15 (15.2)	22 (22.2)	
Bulky lymph nodes	44 (44.0)	117 (34.8)		44 (44.4)	32 (32.3)	
Both	41 (41.0)	87 (25.9)		40 (40.4)	45 (45.5)	

PSM, propensity score matching; FLEEOX, 5-Fu, leucovorin, etoposide, epirubicin and oxaliplatin; XELOX, capecitabine and oxaliplatin

### Tumor response to NAC

The results showed advantageous tumor response to NAC in FLEEOX group (*P*=0.026) (Table 2). 13 (13.1%) patients achieved complete response (CR) in FLEEOX group, which was far more than in XELOX group (3 cases, 3%). The RR was 80.8% and the disease control rate (DCR) was 89.9% in FLEEOX group, whereas in XELOX group, the RR and the DCR were 68.7% and 84.8%, respectively. Therefore, the results suggested a higher RR (*P*=0.018) in FLEEOX group.

**Table 2:** Tumor response to NAC in the two groups after PSM

Variable	FLEEOX (n=99) (%)	XELOX (n=99) (%)	*P*
Response to NAC			**0.026**
CR	13 (13.1)	3 (3.0)	
PR	67 (67.7)	65 (65.7)	
SD	9 (9.1)	16 (16.2)	
PD	10 (10.1)	15 (15.2)	
RR (CR plus PR)	80 (80.8)	68 (68.7)	**0.018**
DCR (CR plus PR plus SD)	89 (89.9)	84 (84.8)	0.196

PSM, propensity score matching; FLEEOX, 5-Fu, leucovorin, etoposide, epirubicin and oxaliplatin; XELOX, capecitabine and oxaliplatin; CR, complete response; PR, partial response; SD, stable disease; PD, progression disease; RR, response rate; DCR, disease control rate

### Surgical characteristics and pathological response

Finally, 80 cases in FLEEOX group and 63 in XELOX group received radical resection, and the radical resection rate in the front group was dramatically higher (80.8% vs. 63.6%, *P*<0.001) (Table 3). R0 surgical rate was also spotted higher in FLEEOX group (69/80, 86.3% vs. 38/63, 60.3%, *P*<0.001). Meanwhile, more grade 3 pathological response occurred in FLEEOX group (11/80, 13.8% vs. 3/63, 4.8%, *P*<0.001). The mean number of dissected lymph nodes in FLEEOX group was less than XELOX group (14.37±6.24 vs. 20.55±10.96, *P*<0.001), whereas the mean number of positive lymph nodes was closed in the both groups (4.23±4.52 vs. 4.89±6.47, P=0.473). The rates of pathological T and N classification were similar between the two groups (*P*=0.409 and 0.061, respectively).

**Table 3:** The characteristics of surgery and pathological response to NAC for the patients received radical surgery after PSM

Variable	FLEEOX (n=80) (%)	XELOX (n=63) (%)	*P*
Patients received surgery			
Radical surgery	80 (80.8)	63 (63.6)	**<0.001**
Palliative surgery	8 (8.1)	16 (16.2)	**<0.001**
No surgery	11 (11.1)	20 (20.2)	
Pathological response			
Grade 3	11 (13.8)	3 (4.8)	**<0.001**
Grade 2	45 (56.2)	38 (60.3)	
Grade 1	24 (30.0)	22 (34.9)	
Residual tumor classification			
R0	69 (69.7)	38 (38.4)	**<0.001**
R1	7 (7.07)	12 (12.1)	
R2	4 (4.04)	13 (13.1)	
TDLNs (mean±SD)	14.37±6.24	20.55±10.96	**<0.001**
Positive LNs (mean±SD)	4.23±4.52	4.89±6.47	0.473
Pathological T classification			0.409
ypT0	11 (13.8)	4 (6.3)	
ypT1	15 (18.8)	7 (11.1)	
ypT2	14 (17.5)	15 (23.8)	
ypT3	16 (20.0)	18 (28.6)	
ypT4a	21 (26.3)	16 (25.4)	
ypT4b	3 (3.8)	3 (4.8)	
Pathological N classification			0.061
ypN0	20 (25.0)	18 (28.6)	
ypN1	20 (25.0)	19 (30.2)	
ypN2	23 (28.8)	9 (14.3)	
ypN3a	8 (10.0)	14 (22.2)	
ypN3b	9 (11.2)	3 (4.8)	

PSM, propensity score matching; FLEEOX, 5-Fu, leucovorin, etoposide, epirubicin and oxaliplatin; XELOX, capecitabine and oxaliplatin; TDLNs, total dissected lymph nodes; LNs, lymph nodes

### Chemotherapy-related toxicity

Overall, no chemotherapy-related deaths and no dropping out of treatment due to adverse events were spotted (Table 4). But, patients in FLEEOX group experienced higher incidence of leukocytopenia (17.2% vs. 7.1%, *P*=0.024), nausea (17.2% vs. 6.1%, *P*=0.012) and vomiting (22.2% vs. 10.1%, *P*=0.016). The overall chemotherapy-related toxicity was dramatically higher in FLEEOX group than XELOX group (71.7% vs. 35.4%, *P*<0.001).

**Table 4:** Grade ¾ adverse events in the patients after PSM

Variable	FLEEOX (n=99) (%)	XELOX (n=99) (%)	*P*
Overall toxicity	71 (71.7)	35 (35.4)	**<0.001**
Related deaths	0 (0%)	0 (0%)	NA
Aborting of NAC	0 (0%)	0 (0%)	NA
Hematological			
Leukocytopenia	17 (17.2)	7 (7.1)	**0.024**
Thrombocytopenia	9 (9.1)	7 (7.1)	0.398
Non-hematological			
Nausea	17 (17.2)	6 (6.1)	**0.012**
Vomiting	22 (22.2)	10 (10.1)	**0.016**
Diarrhea	4 (4.0)	4 (4.0)	0.640
Hepatic inadequacy	2 (2.0)	1 (1.0)	0.500
Renal dysfunction	0 (0.0)	0 (0.0)	-

PSM, propensity score matching; FLEEOX, 5-Fu, leucovorin, etoposide, epirubicin and oxaliplatin; XELOX, capecitabine and oxaliplatin; NA, no available; NAC, neoadjuvant chemotherapy

### Overall survival (OS)

**Figure 2:**
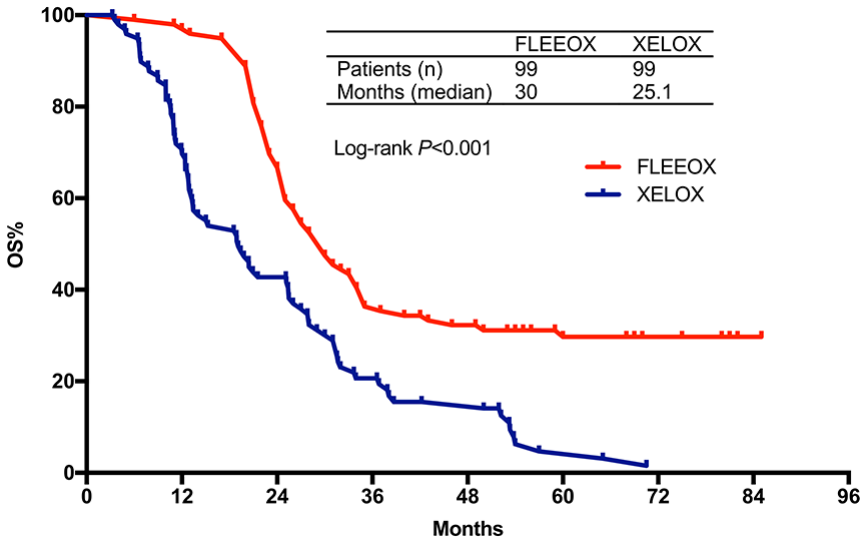
Overall survival according to treatment

**Figure 3:**
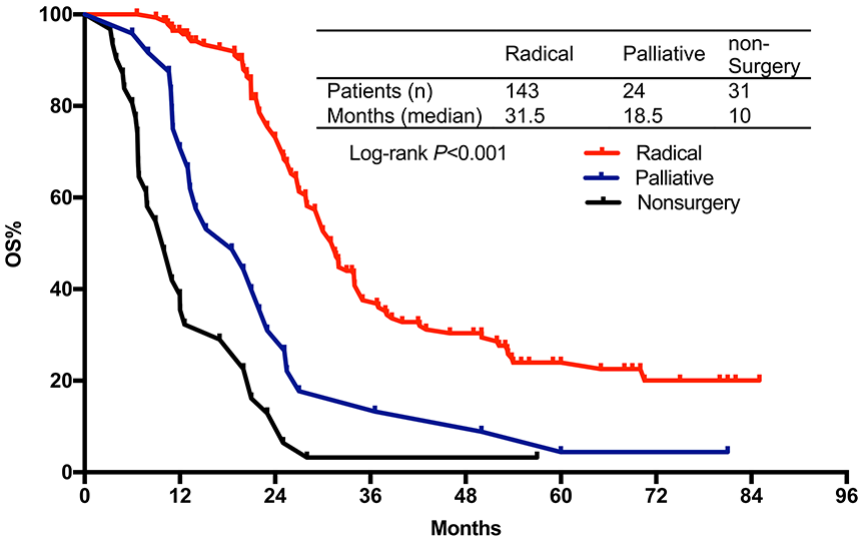
Overall survival stratified by CR+PR vs. SD+PD

**Figure 4:**
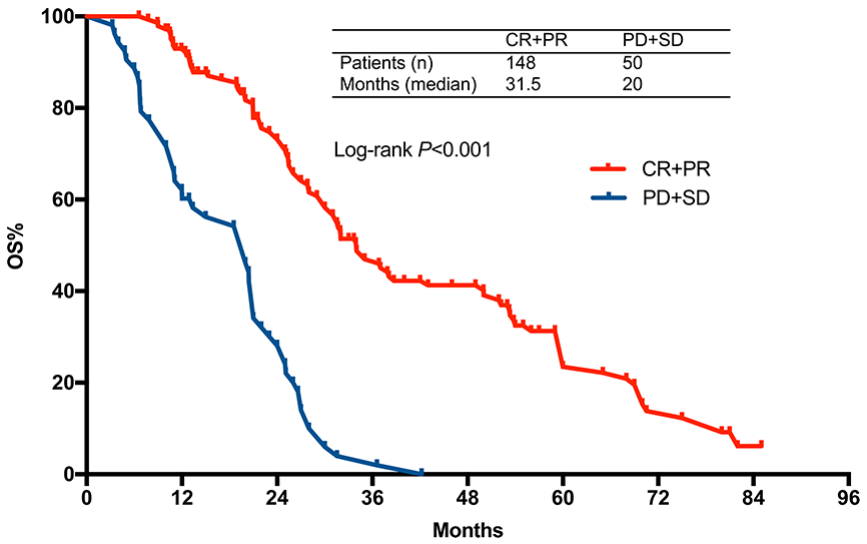
Overall survival stratified by radical resection vs. palliative surgery vs. non-surgery

After a median follow-up of 18 months (range 3.2-85 months), 150 patients (69 in FLEEOX group and 81 in XELOX group) passed away. The median OS time was 30.0 months (95% CI 25.5-34.5) in FLEEOX group and 25.1 months (95% CI 19.3-30.8) in XELOX group (*P*<0.001, Figure 2). Subgroup analysis showed that the patients who received radical surgery had a dramatically longer median OS time than other patients (Radical surgery: 31.3 months vs. Palliative surgery: 18.5 months vs. Non-surgery: 10 months, *P*<0.001, Figure 3). Tumor response had an essential influence on the OS time. Patients who achieved CR or PR had a longer median OS time than patients who achieved SD or PD (31.5 months vs. 20 months, *P*<0.001, Figure 4).

## DISCUSSION

The optimal regimen of NAC for patients with initially unresectable AGC is still a matter of debate. The present study indicated that FLEEOX showed more advantages in terms of RR, radical resection rate, R0 resection rate and OS as compared with XELOX. But a higher overall chemotherapy-related toxicity was also observed in FLEEOX, although none of the patients dropped out of the treatment because of chemotherapy-related toxicity.

In many clinical trials, the RR to NAC for local AGC patients remains around 60%[18–20]. In the study conducted by Nashimoto A et al., S-1 combined cisplatin to treat local AGC patients offered 62.5% of RR [18]. In addition, a better partial RR (77.8%) has been reported in a phase I clinical trial [21], which evaluated the efficacy of FOLFOX4 (oxaliplatin/leucovorin/fluorouracil) for locally unresectable AGC patients. But the sample size in this study was very small (n=9). Our results indicated that RR was 80.8% in FLEEOX group, whereas the RR in XELOX group was 63.6% which was similar to other studies. The high RR in FLEEOX group might be attributed to several reasons. First, the five-drug combination regimen used in this study may be more efficient than two- or three-drug therapies. The mixture of chemotherapeutic agents with different drug mechanisms may offer a combined effect to influence various synthesis procedures in tumor cells and improve the tumor response to NAC. For example, oxaliplatin and epirubicin selectively inhibit the synthesis of deoxyribonucleic acid (DNA) and their cytotoxicity is cell-cycle nonspecific. At high concentration of these drugs, cellular RNA and protein syntheses are also suppressed. Furthermore, etoposide inhibits DNA topoisomerase II, thereby inhibiting DNA re-ligation and affecting mainly the S and G2 phases of tumor cells. Second, the chemotherapy approach via intra-arterial injection increases the regional pharmaceutical concentration and strengthens local effects on the primary tumor and regional metastatic lymph nodes. A pilot study in Japan [22], which based on the same theory as our protocol that the aggressive regional treatment may improve the therapeutic efficacy, showed a high RR of 80% by the addition of local radiotherapy to oral administration of S-1 for gastric cancer patients with severe local infiltration and metastasis. Third, the hepatic first-pass effect of chemotherapeutic drugs, which metabolizes most of the agents, can be partially avoided via intra-arterial administration.

A high RR usually brings satisfactory radical resection rate and R0 resection rate. In the present study, we found that radical resection and R0 rates were 80.8% and 69.7% in FLEEOX group, respectively, which are higher than other studies. A phase II study [12] indicated that twenty-seven initially unresectable AGC patients treated with S-1 plus cisplatin had 63.0% response rate and 48% R0 rate, and another research [10] showed that R0 rate was 63%, in which DXP (docetaxel, cisplatin, and capecitabine) was used as NAC for forty-nine such cases. We also determined that patients could have a survival benefit from radical resection after response to the NAC. Median OS time could be prolonged from 10 to 31.5 months when conversion chemotherapy and radical resection were accomplished, and this result was similar to the previous study [12]. Patients in FLEEOX group had longer median OS time than XELOX group (30 vs. 25.1 months, *P*<0.001) because of a higher radical resection rate and better pathological response, which indicated that the front regimen had more efficacy in patients with initially unresectable AGC. Meanwhile, the radiological response could predict that patients who obtained CR or PR would have better OS than these achieved stable disease (SD) or progressive disease (PD) (31.5 vs. 20 months, *P*<0.001). This means that the response to NAC may predict survival before curative resection of gastric cancer.

Our results also indicated that fewer lymph nodes were harvested in FLEEOX group than that in XELOX group. The possible reason is that more bulky lymph nodes and/or merging of metastatic lymph nodes occurred in FLEEOX group (Table 1). For example, metastatic No. 7, No.8 and No.9 station lymph nodes often merge and encompass the celiac artery as one reason for unresectable AGC. Even after NAC, this combination of metastatic lymph nodes usually cannot be divided and will be considered and reported as a signal metastatic lymph node by pathologists during pathological evaluation. So, the count of harvested lymph nodes in FLEEOX group was less than the actual number when statistical analysis was performed, although the same D2 radical resection was used in the both groups.

In addition, it was apparently known that more aggressive therapy containing multi-drugs caused more adverse events. Our results indicated that NAC-related toxicity, especially in leukocytopenia, nausea and vomiting, tended to be more frequent in FLEEOX group than XELOX group as well the results from other studies [23]. The high frequency of overall chemotherapy-related toxicity in FLEEOX group was ascribed to the high regional concentration of drugs and local irritant effect. But during the period of study, none of the patients quit the treatment for intolerable chemotherapy-related toxicity. And most of the toxicity including leukocytopenia, nausea and vomiting could be alleviated by granulocyte stimulating factor or antiemetic drugs. In another hand, the intra-arterial approach also helped reduce the drug dosages without affecting the therapeutic efficacy. And this could partially explain why the systemic adverse reaction was not increased in FLEEOX regimen, thrombocytopenia, hepatic inadequacy and renal dysfunction, for instance.

Since it is a retrospective study, there are some limitations that cannot be avoided. First, non-randomized and unblinded setting may have a great chance producing selection bias. But, PSM used in this study may well balance the basic characteristics between the two groups. Second, PSM is usually used in large sample studies to control for pre-group differences. In smaller sample size studies, such attrition leaves too few cases for meaningful analysis. But in our study, there were 99 cases in each group after matching which means few subjects missing in the experimental group. Furthermore, Pirracchio et al. indicated that even in the case of small study samples or low prevalence of treatment, PSM can yield correct estimations of treatment effect unless the true confounders and the variables related only to the outcome are not included in the PS model. And no substantial increasing in the Type I error rate was observed as the sample size decreased from 1,000 to 40 subjects.

In conclusion, our research suggested that initially unresectable AGC patients can obtain more benefits from FLEEOX regimen. The more aggressive treatment including five chemotherapeutic drugs would improve RR, radical resection rate, R0 resection rate and OS. In addition, FLEEOX produces more toxicity effects when compared to XELOX, although no chemotherapy-related deaths and aborting of NAC were observed. Further randomized clinical trials about FLEEOX regimen are necessary.

## PATIENTS AND METHODS

### Patients and pretreatment evaluation

This retrospective study was conducted at a single institution. From January 2004 to December 2012, patients with initially unresectable AGC who received NAC were reviewed from our database. As opposed to early-stage gastric cancer, AGC encompasses locally advanced and/or lymph node metastasis. And based on TNM Classification of malignant tumors 7^th^ (TNM 7^th^) [24], AGC in this study includes T3-4, N1-3 and M0. All patients were enrolled according to the following eligibility criteria: 1) histologically proven gastric cancer, 2) evaluated as initially unresectable AGC by abdominal enhanced computed tomography (CT) scan, 3) have signed informed consent before the beginning of treatment, 4) no prior anti-tumor therapy, 5) age 20-70 years old, 6) Karnofsky performance status (KPS) >70, 7) no serious concomitant diseases that make survival period less than 5 years.

Patients are considered as initially unresectable AGC when one or more of the following criteria exist: 1) primary cancer directly infiltrates any one or more of the adjacent structures such as pancreatic head, hepatoduodenal ligament, abdominal main artery, and proximal segment of splenic artery, 2) bulky lymph nodes (larger than 3 cm) or merging of metastatic lymph nodes encompassing vital blood vessels: celiac artery, hepatic artery, splenic artery root, hepatoduodenal ligament, and near portal segment of splenic vein.

Exclusion criteria included: 1) the PLNM by CT scan or left supraclavicular lymph nodal metastasis by biopsy, 2) peritoneal dissemination, 3) other organs metastasis, such as liver and lung, 4) serious or uncontrolled systemic diseases, 5) chemotherapy drug allergies, 6) pregnant or lactating, and 7) with other malignancies contemporaneously, 8) receiving other regimens neither FLEEOX or XELOX. The clinical stage was determined by the TNM Classification of malignant tumors 7^th^ (TNM 7^th^) [24].

### Propensity score matching

The propensity score, as defined by Rosenbaum and Rubin [25], is the individual probability of receiving the treatment of interest conditional on the observed baseline covariates. It has been demonstrated that, within the strata of subjects matched on the propensity score, distributions of these covariates tend to be similar between treated and untreated. In short, PSM was developed to investigate causal relationships between therapeutic protocols and outcomes in a retrospective study other than a randomized controlled trial [26, 27].

In this study, PSM was used to generate a matched pair of cases to compare the clinical efficacy between patients receiving FLEEOX and XELOX. Propensity scores were estimated based on age, gender, tumor location, differentiation, clinical T classification, clinical N classification, and cause of unresection. One-to-one matching without replacement was performed using a 0.05 caliper width [28]. And the score-matched pairs were used in subsequent analyses.

### Neoadjuvant chemotherapy

FLEEOX: 5-Fu (370 mg/m^2^) and leucovorin (200 mg/m^2^) were administered by intravenous infusion on day 1–5. Intra-arterial administration of etoposide (80 mg/ m^2^), oxaliplatin (80 mg/m^2^), and epirubicin (30 mg/m^2^) was performed by Seldinger method on day 6 and 20, the catheter was inserted through femoral artery into the celiac artery and the chemicals were injected initially at relatively high doses, followed by 14 days’ rest.

The blood vessel to inject the drugs was selected by experienced radiologists according to the angiographic results. Usually, chemicals were injected into the left gastric artery for cancer of the upper and middle stomach, while for cancer at the lower stomach the right gastroepiploic artery was selected. Drugs were injected in the order of oxaliplatin, etoposide, and epirubicin, and each injection took 5 min.

XELOX: Intravenous administration of oxaliplatin (130 mg/m^2^) was performed on day 1 and capecitabine was taken orally (1000mg/m^2^) twice a day on day 1 to 14 of a 21-day cycle.

### Evaluation of tumor response and chemotherapy-related toxicity

After two cycles of NAC, the tumor response to chemotherapy was evaluated using an abdominal and pelvic enhanced CT scan by two experienced radiologists, according to the Response Evaluation Criteria in Solid Tumors (RECIST) guideline 1.1 [29]. CR was defined as clinical complete regression of the disease. More than 30% reduction in maximum transverse diameter of the primary tumor was defined as PR, while more than 20% increase in maximum transverse diameter of the primary tumor or the appearance of new lesions was considered as PD. Other cases were included into SD. Radical resection (R0) was determined the operation leaving no macroscopical or microscopical tumor behind. All the chemotherapy-related adverse events during NAC were recorded daily and were evaluated according to National Cancer Institute Common Toxicity Criteria (NCI-CTC) version 3.0 [30].

### Surgical treatment and pathological response

Resectability was assessed by a multidisciplinary team (MDT) after two courses of NAC. Cases with unresectable tumors continued NAC with resectability evaluation every two cycles for a maximum duration of six cycles or until progression. The patients identified as resectable by MDT would receive open surgery. The final surgical procedure would be determined by surgeons during surgery. Patients who were found with unresectable encompassing of vital organs and/or blood vessels during surgery would receive palliative treatment, while others would be given an extended lymph node resection (D2) plus gastrectomy. Surgical specimens were assessed histologically, and the pathological response was evaluated according to the histological criteria of JCGC (Japanese classification of gastric carcinoma, 3rd English edition) [31]. Grade 0, no effect; grade 1, slight effect (grade 1a, viable tumor cells occupy more than 2/3 of the entire cancer area; grade 1b, viable tumor cells remain in more than 1/3 but less than 2/3 of the entire cancer area); grade 2, considerable effect (viable tumor cells remain in less than 1/3 of the entire cancer area); and grade 3, complete response (no viable tumor cells remain).

### Postoperative treatment and follow-up

After surgical treatment, patients were given six cycles of XELOX as adjuvant chemotherapy no matter what the NAC regimen was. Patients who could not undergo a radical resection received other chemotherapy regimens and/or best supportive care. All patients were followed up regularly. Tumor markers including serum carcinoembryonic antigen (CEA) and CA19-9 were examined every 3 months. Chest X-ray and abdominal/pelvic were examined every 6 months. Upper gastrointestinal endoscopy was conducted each year.

### Statistical analysis

All analyses were performed by using SPSS version 24.0 for MAC (SPSS Inc., Chicago, IL, USA) and GraphPad Prism version 7.0 for MAC (GraphPad Software Inc., San Diego, CA, USA). The chi-squared test and Student’s t-test were used for comparisons between the two groups. PSM was applied with a caliper 0.05. Kaplan-Meier analysis with log-rank testing was used for OS. Continuous variables are expressed as mean±standard deviation. *P* value <0.05 was considered statistically significant.

## Abbreviations

FLEEOX: 5-Fu, leucovorin, etoposide, oxaliplatin, and epirubicin
XELOX: capecitabine and oxaliplatin
NAC: neoadjuvant chemotherapy
AGC: advanced gastric cancer
OS: overall survival
ECF: epirubicin, cisplatin and continuous 5-fluorouracil
EAP: etoposide, doxorubicin, and cisplatin
PSM: propensity score matching
PLNM: para-aortic lymph nodal metastases
RR: response rate
CR: complete response
PR: partial response
PD: progressive disease
SD: stable disease
DNA: deoxyribonucleic acid
